# The Antihistamine Astemizole Potentiates the Antitumor Efficacy of Sorafenib in Hepatocellular Carcinoma by Suppressing Proliferation, Metastasis, and Angiogenesis

**DOI:** 10.3390/cimb48050451

**Published:** 2026-04-26

**Authors:** Yixuan Zhang, Xin Chen, Xuting Yang, Peiyu Wang, Xiaoliang Zhang, Dexin Kong, Ran Wang

**Affiliations:** 1 Tianjin Key Laboratory of Technologies Enabling Development of Clinical Therapeutics and Diagnostics, School of Pharmacy, Tianjin Medical University, Tianjin 300070, China; zhangyixuan@tmu.edu.cn (Y.Z.); chenxin1234@tmu.edu.cn (X.C.); xuting@tmu.edu.cn (X.Y.); peiyuwang@tmu.edu.cn (P.W.); zhangxiaoliang12@tmu.edu.cn (X.Z.); 2 International Joint Laboratory of Ocular Diseases (Ministry of Education), Tianjin Medical University, Tianjin 300070, China; 3 Key Laboratory of Immune Microenvironment and Diseases (Ministry of Education), Tianjin Medical University, Tianjin 300070, China

**Keywords:** sorafenib, astemizole, hepatocellular carcinoma, proliferation, metastasis, angiogenesis, Eag1, ERK/MAPK

## Abstract

Hepatocellular carcinoma (HCC) is a highly aggressive malignancy with a poor prognosis. While sorafenib serves as the first-line therapy for advanced HCC, its efficacy is frequently hampered by side effects and the development of drug resistance, necessitating the development of novel agents to enhance HCC sensitivity to sorafenib. In this study, we demonstrate that the antihistamine astemizole significantly enhanced the antitumor efficacy of sorafenib in HCC cell lines. This combination treatment cooperatively inhibited HCC cells’ proliferation and induced cell cycle arrest at the G1 phase, as evidenced by decreased cyclin D1 and p-Rb levels and increased p27 expression. Furthermore, the combination of astemizole and sorafenib synergistically inhibited HCC cells’ migration, invasion, and adhesion. It also reduced F-actin polymerization and the expression of metastasis-regulating proteins, including p-Integrinβ1, FAK, and MMP1. Additionally, the combination treatment suppressed tube formation in HUVECs, accompanied by downregulation of HIF-1α and reduced VEGF secretion. Co-inhibition of Eag1 and the ERK/MAPK signaling pathway may underlie the enhanced anti-HCC effects of sorafenib by astemizole. Collectively, these findings indicate that astemizole significantly enhanced the antitumor activity of sorafenib by inhibiting proliferation, metastasis, and angiogenesis in HCC cells, suggesting its potential as a promising adjuvant to improve sorafenib-based therapy in HCC.

## 1. Introduction

Liver cancer is a leading digestive system malignancy worldwide, ranking as the sixth most common malignancy and the third leading cause of cancer-related mortality [1]. Hepatocellular carcinoma (HCC) is the predominant type of liver cancer, accounting for ~90% of all primary liver cancer cases [2]. While surgical resection can be curative for early-stage disease, most patients are diagnosed at an advanced stage due to a lack of early symptoms, at which point invasion and metastasis drive poor outcomes [3]. Particularly, the development of extrahepatic metastases is associated with a particularly poor prognosis (median survival: 4.9–7 months; 1-year survival <35%) [4,5]. Systemic pharmacotherapy, including chemotherapy and molecularly targeted agents, therefore forms the cornerstone of treatment for advanced, metastatic HCC [6].

Sorafenib is an orally administered multi-target tyrosine kinase inhibitor approved by the U.S. Food and Drug Administration (FDA) for the treatment of patients with HCC [7]. It suppresses tumor cell proliferation primarily by blocking the activity of Raf-1 and B-Raf kinases within the Ras/Raf/MEK/ERK signaling pathway. Additionally, sorafenib targets receptor tyrosine kinases such as c-Kit, VEGFR-2, VEGFR-3, and PDGFR-β, thereby suppressing tumor angiogenesis [8]. Despite demonstrating clinical efficacy in HCC, the survival benefit of sorafenib remains modest. Data from phase III multicenter randomized controlled trials indicate that sorafenib extends median overall survival by only approximately 2–3 months compared with placebo (10.7 vs. 7.9 months) [7,9,10]. Moreover, treatment-related toxicities and the emergence of adaptive resistance within approximately six months in some patients frequently compromise therapeutic efficacy [3]. Therefore, there is an urgent need to develop optimized treatment strategies to improve the clinical efficacy of sorafenib-based therapy.

Astemizole is a long-acting, non-sedating antihistamine clinically used for allergic diseases through antagonism of the histamine H1 receptor [11]. Beyond its classical antihistaminic activity, astemizole has been identified as a blocker of the Eag (ether-à-go-go) potassium channel family, exerting its inhibitory effect by selectively binding to and suppressing the activity of Eag1 [12]. Under physiological conditions, Eag1 is predominantly expressed in the central nervous system and shows low expression in most non-neuronal tissues [13]. However, accumulating evidence indicates that Eag1 is aberrantly overexpressed in various malignancies, including HCC, where its expression correlates with tumor progression and poor prognosis [14,15,16]. Additionally, because astemizole crosses the blood–brain barrier less readily than other antihistamines, it is less likely to induce central nervous system suppression at standard doses, thereby enabling relatively selective targeting of tumor-associated Eag1 for antitumor intervention [17]. Consistent with this notion, astemizole has been demonstrated to exert broad-spectrum anti-proliferative and pro-apoptotic effects across multiple malignancies, effectively suppressing the growth of leukemia and cervical cancer cells, as well as Eag1-expressing transformed models [18]. Beyond its efficacy as a monotherapy, emerging evidence underscores the superior antineoplastic potential of astemizole-based combination regimens. For instance, dual targeting of the oncogenic Eag1 channel by co-administering astemizole with calcitriol synergistically enhances tumor growth inhibition in breast cancer [19]. Furthermore, this combinatorial advantage extends to other aggressive solid tumors; notably, integrating astemizole with the EGFR inhibitor gefitinib yields a significantly augmented therapeutic response in human lung cancer compared to single-agent treatments [20].

Drug repositioning offers a strategic advantage in oncology by leveraging approved agents for new indications, thereby accelerating development and reducing costs while building upon established safety profiles. In this study, we for the first time investigated the combination of the antihistamine astemizole with the first-line targeted agent sorafenib in HCC cells, aiming to elucidate their synergetic effects on cancer proliferation and metastasis and the underlying molecular mechanisms, thus offering a potential strategy to enhance the therapeutic efficacy of sorafenib.

## 2. Materials and Methods

### 2.1. Reagents and Antibodies

Sorafenib and astemizole were purchased from MedChemExpress (Monmouth Junction, NJ, USA). The Annexin V-FITC/PI apoptosis detection kit was purchased from Meilunbio (Dalian, China). The following antibodies were used: CyclinD1 (Cell Signaling Technology, Danvers, MA, USA, 2978), P-Rb (Cell Signaling Technology, 9307), p27 (Cell Signaling Technology, 3688), Eag1 (Abcam, Cambridge, UK, ab247687), p-ERK (Cell Signaling Technology, 5683), ERK (Abclonal, Wuhan, China, A4782), p-integrinβ1 (Abcam, ab5189), MMP1 (Abcam, ab38929), FAK (Cell Signaling Technology, 13009), HIF-1α (Cell Signaling Technology, 36169), VEGFA (Proteintech (Wuhan Sanying Biotechnology), Wuhan, 66828), GAPDH (Cell Signaling Technology, 2118), Anti-rabbit IgG Antibody (Cell Signaling Technology, 7074) and Anti-mouse IgG Antibody (Cell Signaling Technology, 7076).

### 2.2. Cell Culture

Human HCC cell lines Huh7 and HepG2 were purchased from the cell bank of the Chinese Academy of Sciences (Shanghai, China). Human umbilical vein endothelial cells (HUVECs) were obtained from Lifeline Cell Technology (Frederick, MD, USA). All cells were cultured in Dulbecco’s Modified Eagle Medium (Biological Industries, Beit HaEmek, Israel) supplemented with 10% fetal bovine serum (Biological Industries), 100 U/mL penicillin, and 100 μg/mL streptomycin. Cells were maintained at 37 °C in a humidified incubator containing 5% CO_2_. Routine passaging was performed every 2 days, and cells were used within 20 passages.

### 2.3. Cell Viability Assay

Cell viability was assessed using the MTT assay as previously described [21]. Cells were seeded into 96-well plates and treated with the indicated drugs for 48 or 72 h. Subsequently, 20 μL of MTT solution (4 mg/mL; Beyotime Biotechnology, Shanghai, China) was added per well for a 4 h incubation. After medium removal, formazan crystals were dissolved in 150 μL DMSO, and absorbance was measured at 490 nm using an iMark microplate reader (Bio-Rad, Hercules, CA, USA). The half-maximal inhibitory concentration (IC_50_) values were calculated via GraphPad Prism (version 9.00).

### 2.4. Drug Synergy Assay

Drug synergism was evaluated using Chou-Talalay’s method [21]. Following 72 h of single or combined treatment with astemizole and sorafenib for 3 days, cell growth inhibition data were analyzed via CalcuSyn software (version 2.1). Combination index (CI) values were calculated to define drug interactions: CI < 1 (synergism), CI = 1 (additive effect), and CI > 1 (antagonism).

### 2.5. Colony Formation Assay

Colony formation was evaluated in Huh7 and HepG2 cells as previously described [21]. Cells (500 cells/well) were seeded in 6-well plates and treated with the indicated drugs for 14 days (medium refreshed every 3 days). Following incubation, colonies were fixed with 4% paraformaldehyde for 15 min and stained with 0.5% crystal violet (Solarbio, Beijing, China) for 30 min. Surviving colonies (>10 cells) were counted for statistical analysis.

### 2.6. Cell Cycle Analysis

Cell cycle distribution was analyzed as previously described [21]. Huh7 and HepG2 cells were seeded in 6-well plates at a density of 1 × 10^5^ cells/mL. Following overnight attachment and 72 h of drug treatment, harvested cells were resuspended in 100 μL PBS and stained with 50 μg/mL propidium iodide (PI) for 1 h in the dark. Samples were subsequently acquired and analyzed using an Accuri C6 flow cytometer (BD Biosciences, Franklin Lakes, NJ, USA).

### 2.7. EdU (5-Ethynyl-2′-Deoxyuridine) Staining

EdU staining was performed as previously described [22]. Huh7 and HepG2 cells were seeded onto sterile glass coverslips placed in 12-well plates at a density of 4 × 10^4^ cells/mL. After overnight attachment, cells were treated with astemizole or sorafenib alone, or in combination. After 72 h of treatment, 1 μL of EdU working solution (10 μM; Beyotime Biotechnology) was added to each well and incubated for an additional 2 h. Cells were subsequently fixed with 4% paraformaldehyde, permeabilized with 0.3% Triton X-100, and blocked with 3% BSA. The Click-iT reaction was carried out for 30 min at room temperature in the dark. After washing with PBS, cell nuclei were counterstained with Hoechst 33342 for 10 min under light-protected conditions. Coverslips were mounted, and fluorescence images were captured using a BX51 fluorescence microscope (Olympus, Japan). The cell proliferation rate was quantified as the percentage of EdU-positive cells relative to total nuclei using ImageJ software (version 1.53k).

### 2.8. Apoptosis Assay

Cell apoptosis was analyzed as previously described [23]. Huh7 and HepG2 cells were seeded into 6-well plates at a density of 1.0 × 10^5^ cells/mL and treated with astemizole or sorafenib alone, or in combination. After 72 h of treatment, cells were harvested and resuspended in 400 μL of 1× binding buffer. Cells were then stained with Annexin V-FITC (2.5 μL) and PI (2.5 μL) for 15 min at room temperature in the dark. The proportion of apoptotic cells was subsequently quantified by flow cytometry using an Accuri C6 flow cytometer (BD Biosciences).

### 2.9. Western Blotting

Western blotting was performed as previously described [23]. Briefly, cells were seeded into 6-well plates and treated with astemizole, sorafenib, or their combination for the indicated durations. Cells were then harvested and lysed using RIPA buffer (Beyotime Biotechnology). Protein concentrations were determined using the Pierce™ BCA protein assay kit (Thermo Fisher Scientific, Waltham, MA, USA). Equal amounts of total protein (50 μg) were separated by 8% or 10% SDS-PAGE and subsequently transferred onto PVDF membranes (Millipore, Billerica, MA, USA). Membranes were blocked with 5% non-fat milk and incubated overnight at 4 °C with the primary monoclonal antibodies, followed by four washes with TBST. The membranes were then incubated with the corresponding HRP-conjugated secondary antibodies for 1 h at room temperature. Protein bands were visualized using an ECL detection system (Bio-Rad) and quantified using ImageJ software (version 1.53k).

### 2.10. Transwell Migration Assay

The migratory ability of HCC cells was evaluated using a Transwell migration assay as previously described [23]. Huh7 and HepG2 cells were suspended and seeded into the upper chambers of transwell inserts (Corning, Corning, NY, USA) at densities of 3 × 10^5^ cells/mL and 8 × 10^5^ cells/mL, respectively. The lower chambers were filled with 600 μL of culture medium supplemented with fetal bovine serum. Astemizole, sorafenib, or their combination was added to both the upper and lower chambers. After incubation for 48 h, non-migrated cells on the upper surface of the membrane were gently removed with PBS. Migrated cells on the lower surface were fixed with 4% paraformaldehyde for 30 min, stained with crystal violet for 15 min, and rinsed with PBS. Images were captured using an inverted microscope (Axio Observer 5, Carl Zeiss, Jena, Germany), and the number of migrated cells was counted using ImageJ software (version 1.53k).

### 2.11. Transwell Invasion Assay

For the invasion assay, matrigel (Corning) was thawed overnight at 4 °C and diluted 1:8 with serum-free medium. A total of 50 μL of diluted matrigel was added to each insert of the transwell chamber and incubated at 37 °C for 5 h to allow gel polymerization. Thereafter, cell seeding, drug treatment, and subsequent detection procedures were performed as described for the transwell migration assay.

### 2.12. Cell Adhesion Assay

The cell adhesion assay was performed as previously described [23]. Huh7 and HepG2 cells were seeded into 12-well plates at a density of 4 × 10^4^ cells/mL and treated with astemizole, sorafenib, or their combination for 48 h. A 96-well plate was pre-coated with 50 μL per well of MSC cell adhesion reagent (Corning) and incubated at 37 °C for 1 h. After blocking with 1% BSA at 37 °C for 1 h and washing twice with serum-free medium, treated cells were seeded into the coated wells and allowed to adhere for 2 h at 37 °C. Non-adherent cells were removed by gentle washing with PBS. Adherent cells were subsequently fixed with 4% paraformaldehyde, stained with crystal violet, and rinsed with PBS. The bound dye was solubilized in 33% acetic acid, and absorbance was measured at 570 nm using an iMark microplate reader (Bio-Rad).

### 2.13. Actin Cytoskeleton Analysis

Huh7 and HepG2 cells were seeded into 12-well plates and treated with astemizole, sorafenib, or their combination for 48 h. After treatment, cells were fixed with 4% paraformaldehyde for 20 min at room temperature and permeabilized with 0.1% Triton X-100 for 10 min, followed by washing with PBS. F-actin was stained with iFluor™ 647-phalloidin (1 μg/mL) working solution for 60 min, and cell nuclei were counterstained with Hoechst (10 μg/mL) for 10 min. After washing with PBS three times, fluorescence images were acquired using a confocal laser scanning microscope (LSM 800, Carl Zeiss,) equipped with iFluor™ 647 (Ex/Em = 650/665 nm) and/or DAPI (Ex/Em = 364/454 nm) filter sets.

### 2.14. Tube Formation Assay

The tube formation assay was performed as previously described [23]. HCC cells were seeded into 6 cm culture dishes at a density of 1 × 10^5^ cells/mL and treated with astemizole, sorafenib, or their combination for 48 h. Conditioned media were subsequently collected, centrifuged at 1000 rpm for 5 min to remove cellular debris, and stored for further use. For the tube formation assay, 96-well plates were precoated with matrigel (BD Biosciences) and incubated at 37 °C for 1 h to allow gel polymerization. HUVECs were harvested and resuspended in the conditioned media at a density of 2 × 10^5^ cells/mL. A total of 100 μL of the cell suspension was added to each matrigel-coated well and incubated at 37 °C. Tube-like structures were observed and imaged after 6 h using an inverted microscope (Axio Observer 5, Carl Zeiss).

### 2.15. Enzyme-Linked Immunosorbent Assay (ELISA)

Huh7 and HepG2 cells were seeded into 6 cm culture dishes at a density of 1 × 10^5^ cells/mL and treated with astemizole, sorafenib, or their combination for 72 h. Following treatment, cell culture supernatants were collected for subsequent analysis. ELISA was performed according to the manufacturer’s instructions (Shanghai Enzyme-linked biotechnology, Shanghai, China). Briefly, 100 μL of assay diluent and 50 μL of standards or cell supernatants were added to each well of a 96-well plate. After incubation for 2 h at room temperature, the plate was washed three times with washing buffer. Subsequently, human HRP-conjugated anti-VEGF antibody was added to each well and incubated for an additional 2 h. After washing, 200 μL of substrate solution was added, and the plate was incubated in the dark for 30 min. The reaction was terminated by adding 50 μL of stop solution, and absorbance was measured at 450 nm using an iMark microplate reader (Bio-Rad).

### 2.16. Statistical Analysis

Statistical analyses were performed using GraphPad Prism software (version 9.00). Data are expressed as the mean ± standard deviation (SD) from three independent experiments. Differences between pairs of mean values were determined by Student’s *t*-test. A *p*-value < 0.05 was considered statistically significant.

## 3. Results

### 3.1. Astemizole Potentiates the Antiproliferative Effect of Sorafenib in HCC Cells

**Figure 1:**
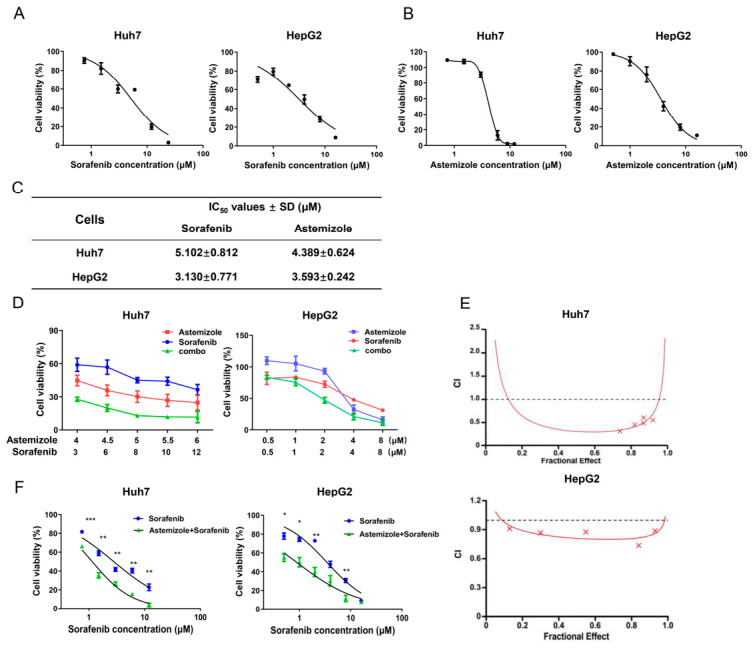
The in vitro antiproliferative activities of astemizole and sorafenib as a single agent or in combination in HCC cells. (**A**,**B**) Dose–response curves of sorafenib (**A**) and astemizole (**B**) in Huh7 and HepG2 cells after 72 h treatment, as assessed by MTT assay. Concentration ranges: for sorafenib, 0.75–24 µM (Huh7) and 0.5–16 µM (HepG2); for astemizole, 0.75–12 µM (Huh7) and 0.5–16 µM (HepG2). (**C**) Half-maximal inhibitory concentration (IC_50_) values of sorafenib and astemizole in the two cell lines. (**D**) Cell viability following treatment with astemizole, sorafenib, or their combination for 72 h was determined by MTT assay. (**E**) Combination index (CI) analysis performed using CalcuSyn software, and the corresponding CI-Fa plots are shown. The horizontal line indicates CI = 1, representing an additive effect. CI values below this line indicate synergism. (**F**) Dose–response curves of sorafenib in the presence of a fixed low-toxicity concentration of astemizole (3.4 µM for Huh7; 1.7 µM for HepG2) after 72 h treatment. Data are presented as mean ± SD (*n* = 3). * *p* < 0.05, ** *p* < 0.01, *** *p* < 0.001 versus the sorafenib monotherapy group.

We first evaluated the antiproliferative effects of sorafenib and astemizole in human HCC Huh7 and HepG2 cells. MTT assays demonstrated that both compounds reduced cell viability in a dose-dependent manner in the two cell lines (Figure 1A,B). The IC_50_ values of sorafenib were 5.102 μM in Huh7 cells and 3.130 μM in HepG2 cells, whereas the IC_50_ values of astemizole were 4.389 μM and 3.593 μM, respectively (Figure 1C). We next investigated whether astemizole could potentiate the antiproliferative activity of sorafenib. Combined treatment with astemizole and sorafenib resulted in a significantly greater reduction in cell viability compared with either agent alone in both Huh7 and HepG2 cells (Figure 1D). To quantitatively assess the interaction between the two compounds, combination effects were analyzed using Chou-Talalay’s method [24]. As shown in Figure 1E and Table 1, the combination index (CI) values were consistently below 1 across ED_50_-ED_90_ in both cell lines, indicating a synergistic interaction between astemizole and sorafenib. Given that astemizole is clinically used as a histamine receptor antagonist for allergic conditions, high doses may pose potential side effects in human tissues. To minimize toxicity while exploring combinatorial efficacy, a subtoxic concentration of astemizole (below IC_20_) was applied in combination with graded doses of sorafenib. Notably, even at this low-toxic concentration, astemizole significantly enhanced the antiproliferative efficacy of sorafenib in both HCC cell lines (Figure 1F).

### 3.2. Combination Treatment with Astemizole and Sorafenib Inhibits DNA Synthesis and Colony Formation in HCC Cells

**Figure 2:**
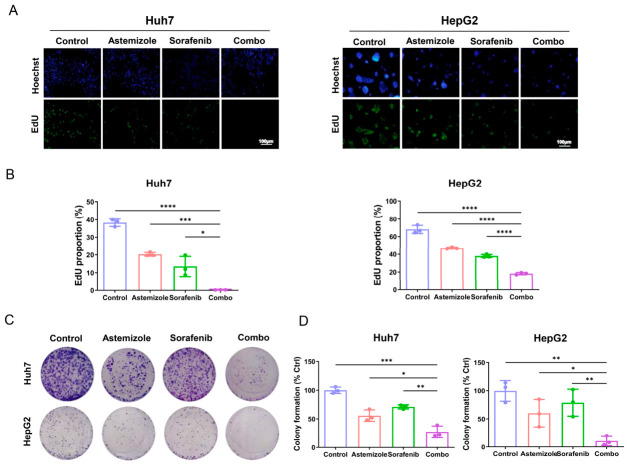
Combination of astemizole and sorafenib suppresses DNA synthesis and colony-forming ability of HCC cells. (**A**) Representative fluorescence images of EdU (green) and Hoechst 33342 (blue) staining in Huh7 and HepG2 cells following 72 h treatment with astemizole and sorafenib (Huh7: 3.4 µM astemizole + 3 µM sorafenib; HepG2: 1.7 µM astemizole + 2 µM sorafenib; the same concentrations were used in subsequent anti-proliferation experiments). Scale bar = 100 µm. (**B**) The percentage of EdU-positive cells was quantified using ImageJ software. (**C**) Representative images of crystal violet-stained colonies after 14 days of treatment with astemizole, sorafenib, or their combination. (**D**) Colony formation efficiency was quantified relative to the control group. Data are presented as mean ± SD (*n* = 3). * *p* < 0.05, ** *p* < 0.01, *** *p* < 0.001, **** *p* < 0.0001.

To further evaluate the effects of combined astemizole and sorafenib treatment on the proliferative capacity of HCC cells, DNA synthesis was assessed using the EdU incorporation assay, a sensitive and quantitative method for measuring cell proliferation [25]. As shown in Figure 2A, the proportion of EdU-positive cells was markedly reduced in the combination treatment group compared with the control and single-agent groups in both Huh7 and HepG2 cell lines. Quantitative analysis further confirmed that the proliferation rate was significantly lower in the combination group than in either monotherapy group (Figure 2B). Subsequently, a colony formation assay was conducted to examine the long-term impact on clonogenic survival. Consistent with the EdU results, combined treatment with astemizole and sorafenib significantly suppressed colony formation in both HCC cell lines compared to either agent alone (Figure 2C,D). Taken together, these findings demonstrate that astemizole markedly enhances the antiproliferative effect of sorafenib by inhibiting DNA synthesis and clonogenic growth in HCC cells, supporting the therapeutic potential of this combination strategy for HCC.

### 3.3. Combination of Sorafenib with Astemizole Enhances G1 Phase Arrest Without Inducing Apoptosis

**Figure 3:**
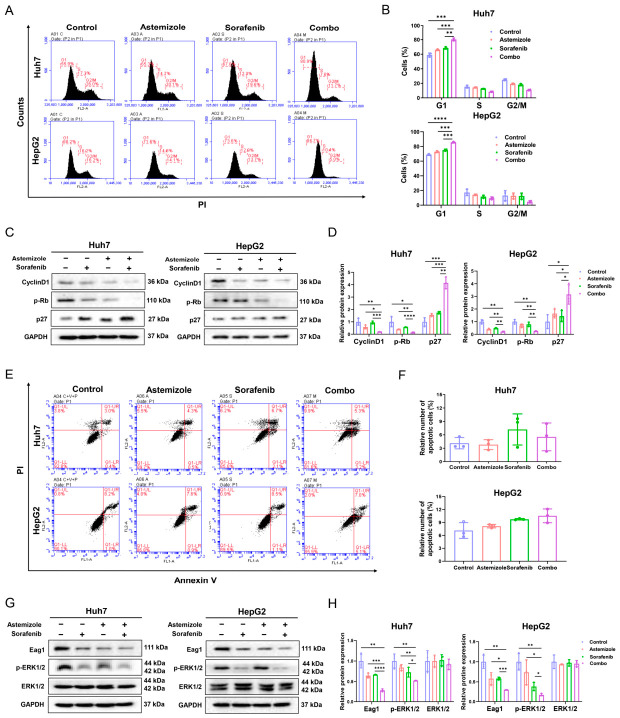
Combined treatment with astemizole and sorafenib synergistically induces G1-phase arrest and inhibits Eag1 and ERK/MAPK signaling in HCC cells. Huh7 and HepG2 cells were treated with astemizole, sorafenib, or their combination for 72 h. (**A**) Cell cycle distribution was analyzed by flow cytometry following PI staining. (**B**) Quantification of the percentages of cells in the G1, S, and G2/M phases. (**C**) Western blot analysis of G1 phase-associated cell cycle regulatory proteins, including Cyclin D1, p-Rb, and p27, with GAPDH used as a loading control. (**D**) Densitometric analysis of the relative expression levels of Cyclin D1, p-Rb and p27. (**E**) Apoptosis was assessed by Annexin V and PI double staining. (**F**) Flow cytometric quantification of apoptotic cell populations, including Annexin V^+^/PI^−^ early apoptotic cells and Annexin V^+^/PI^+^ late apoptotic cells. (**G**) Expression levels of Eag1 and key components of the ERK/MAPK signaling pathway were examined by Western blotting, with GAPDH used as a loading control. (**H**) Densitometric analysis of the relative expression levels of Eag1, p-ERK1/2 and ERK1/2. Data are presented as mean ± SD (*n* = 3). * *p* < 0.05, ** *p* < 0.01, *** *p* < 0.001, **** *p* < 0.0001.

To investigate the mechanism underlying the synergistic activity, the effects of astemizole and sorafenib on cell cycle progression were assessed. Flow cytometric analysis demonstrated that while each agent alone induced a modest increase in the proportion of cells in G1 phase, their combination resulted in a significantly more pronounced G1 arrest in both Huh7 and HepG2 cell lines compared to monotherapies (Figure 3A,B). Consistent with this phenotype, Western blot analysis revealed that combined treatment markedly downregulated Cyclin D1 expression and reduced Rb phosphorylation, while concomitantly upregulating the cyclin-dependent kinase inhibitor p27 in both cell lines (Figure 3C,D). These changes in G1/S checkpoint-associated proteins align with a reinforced G1/S checkpoint blockade. In contrast, apoptosis analysis by Annexin V/PI staining showed no significant increase in apoptotic cell populations following either single or combined treatment (Figure 3E,F), indicating that the synergistic antiproliferative effect is not mediated through apoptosis induction. Collectively, these results demonstrate that combined treatment with astemizole and sorafenib induces a robust G1 phase arrest through modulation of key cell cycle regulators, without eliciting a substantial apoptotic response in HCC cells.

### 3.4. Synergistic Inhibition of Eag1 and ERK/MAPK Signaling by Astemizole and Sorafenib in HCC Cells

Eag1, a voltage-gated potassium channel protein, is frequently overexpressed in multiple malignancies, including HCC, where its elevated expression correlates with unfavorable clinical outcomes [14,26]. Given that astemizole functions as an Eag1 inhibitor, we examined the effects of astemizole and sorafenib, both individually and in combination, on Eag1 expression in HCC cells. As shown in Figure 3G,H, treatment with either astemizole or sorafenib resulted in a reduction in Eag1 expression, with the combined treatment producing a more substantial reduction compared to either agent alone. We further investigated the influence of these treatments on the ERK/MAPK signaling cascade, a critical pathway involved in regulating tumor proliferation, metastasis, and therapy resistance [27]. Sorafenib, a recognized B-RAF inhibitor, has been previously reported to inhibit ERK/MAPK signaling [8]. Consistent with this, treatment with sorafenib alone decreased phosphorylation of ERK, and this inhibitory effect was further enhanced when sorafenib was combined with astemizole (Figure 3G,H). Taken together, these findings demonstrate that astemizole and sorafenib act synergistically to suppress HCC cell proliferation, possibly in part through concurrent downregulation of Eag1 expression and suppression of ERK/MAPK signaling pathway activation.

### 3.5. Combination Treatment with Astemizole and Sorafenib Suppresses Migration, Invasion, and Adhesion of HCC Cells

**Figure 4:**
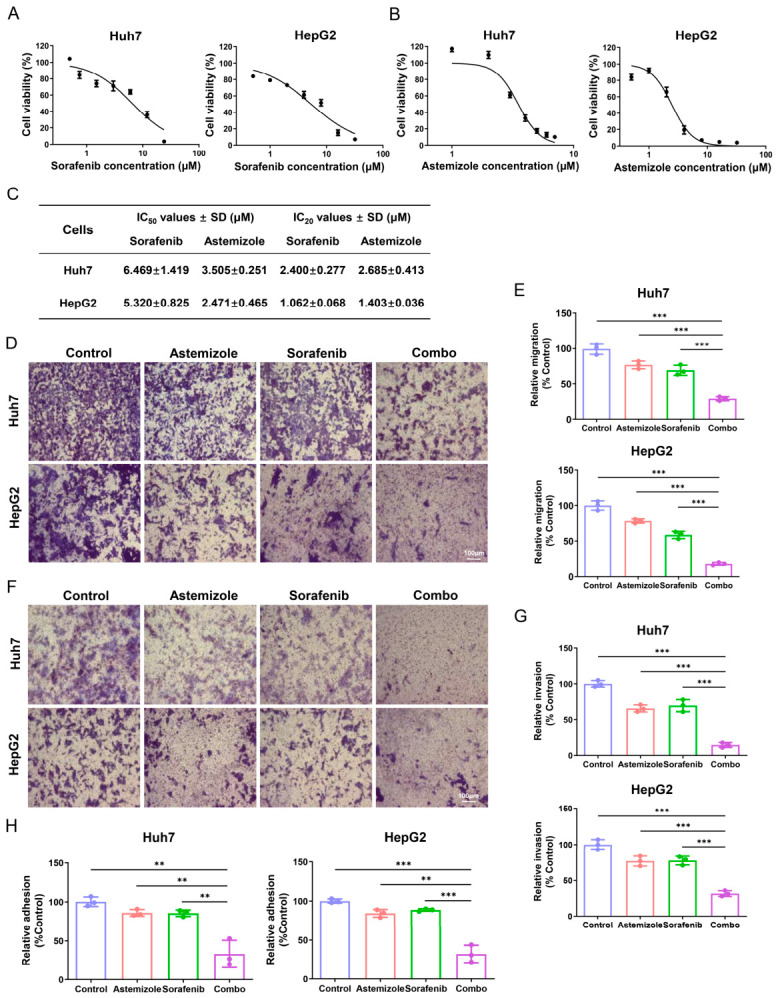
Inhibitory effects of combined astemizole and sorafenib treatment on migration, invasion, and adhesion of HCC cells. (**A**,**B**) Cell viability of Huh7 and HepG2 cells treated for 48 h with increasing concentrations of sorafenib (**A**) or astemizole (**B**), as measured by MTT assay. Concentration ranges: sorafenib, 0.5–24 µM (Huh7) and 0.5–32 µM (HepG2); astemizole, 1–7 µM (Huh7) and 0.5–32 µM (HepG2). (**C**) Summary of IC_50_ and IC_20_ values of astemizole and sorafenib in Huh7 and HepG2 cells. Sub-cytotoxic concentrations (<IC_20_) were used in subsequent metastasis-related assays to exclude the influence of growth inhibition (Huh7: 2.6 µM astemizole + 2.4 µM sorafenib; HepG2: 1.4 µM astemizole + 1 µM sorafenib). (**D**) Representative images of crystal violet-stained cells after transwell migration assay following 48 h treatment with astemizole and/or sorafenib. Scale bar = 100 µm. (**E**) Quantification of migrated cells expressed as a percentage relative to the control group. (**F**) Representative images from matrigel-coated transwell invasion assay after 48 h treatment with astemizole and/or sorafenib. Scale bar = 100 µm. (**G**) Quantitative analysis of cells invading through matrigel-coated membranes. (**H**) Quantification of adhesion cells following 48 h treatment with astemizole and/or sorafenib. Data are presented as mean ± SD (*n* = 3). ** *p* < 0.01, *** *p* < 0.001.

To evaluate the effects of astemizole and sorafenib on HCC cell metastasis, we first assessed cell viability following 48 h of treatment. Drug concentrations with less influence on cell proliferation (below IC_20_) were selected for subsequent experiments in Huh7 and HepG2 cells (Figure 4A–C). In the transwell migration assay, combined treatment with astemizole and sorafenib significantly reduced the number of migrated cells compared to either monotherapy or the control. Quantitative analysis showed that migration rates relative to the control were 76.7%, 69.0%, and 29.0% in Huh7 cells treated with astemizole, sorafenib, or the combination, respectively. Similarly, in HepG2 cells, migration rates were 78.3%, 58.7%, and 18.0% for astemizole, sorafenib, and the combination, respectively (Figure 4D,E). We next examined the effects of astemizole and sorafenib on the invasive capacity of HCC cells using a matrigel-coated transwell assay. The combination of astemizole and sorafenib markedly reduced the number of invaded cells compared to single-agent treatments. In Huh7 cells, invasion rates decreased to 65.7%, 69.7%, and 14.7% of the control following treatment with astemizole, sorafenib, or the combination, respectively. Corresponding rates in HepG2 cells were 77.0%, 77.7%, and 31.7% (Figure 4F,G). Since adhesion to the extracellular matrix (ECM) is critical for metastatic dissemination [28], we further investigated the effect of combined astemizole and sorafenib treatment on cell adhesion. After 48 h of exposure, the combination significantly impaired HCC cell adhesion to fibronectin-coated surfaces, reducing adherent cell numbers to approximately one-third of those in the control group (Figure 4H). Collectively, these results demonstrate that astemizole and sorafenib exert a synergistic inhibitory effect on multiple key steps of the metastatic process in HCC cells, including migration, invasion, and adhesion.

### 3.6. Astemizole and Sorafenib Suppress HCC Cell Migration by Disrupting F-Actin Polymerization and Downregulating Metastasis-Associated Proteins

**Figure 5:**
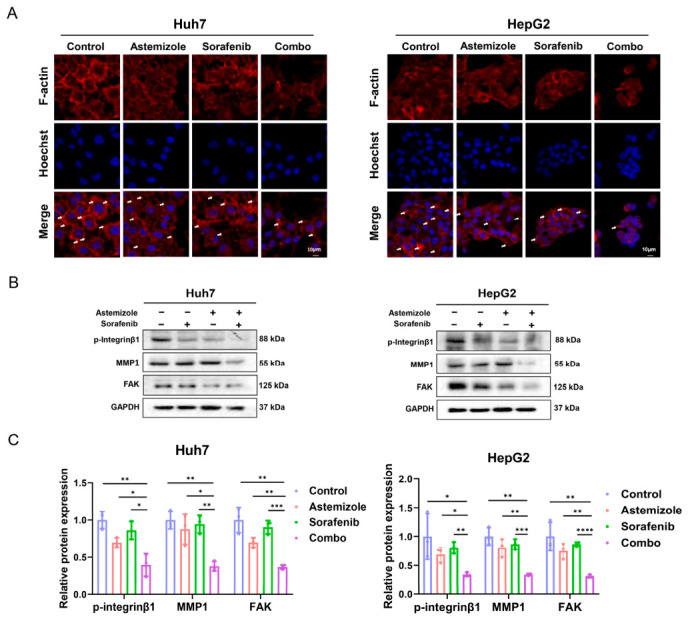
The combination of astemizole and sorafenib disrupts cytoskeletal organization and downregulates metastasis-associated proteins. (**A**) Representative immunofluorescence images of F-actin cytoskeleton in cells treated with astemizole and/or sorafenib for 48 h. F-actin filaments were labeled with phalloidin (red), and nuclei were counterstained with Hoechst 33342 (blue). White arrows indicate membrane protrusions at the cell periphery. Scale bar = 10 μm. (**B**) Western blot analysis of metastasis-related proteins, including p-Integrinβ1, MMP1, and FAK, following treatment with astemizole and/or sorafenib for 48 h. GAPDH was used as a loading control. (**C**) Densitometric analysis of the relative expression levels of p-Integrinβ1, MMP1, and FAK. Data are presented as mean ± SD (*n* = 3). * *p* < 0.05, ** *p* < 0.01, *** *p* < 0.001, **** *p* < 0.0001.

To elucidate the anti-metastatic mechanism of the astemizole and sorafenib combination in HCC, we first analyzed F-actin cytoskeletal organization in Huh7 and HepG2 cells via immunofluorescence staining. F-actin, a core cytoskeletal component, undergoes dynamic polymerization and depolymerization to drive cell motility and contraction, processes essential for migration [29]. Compared with control or single-agent treatments, the combination of astemizole and sorafenib markedly reduced F-actin fluorescence intensity, accompanied by decreased cell size, reduced formation of membrane protrusions, and smoother cell boundaries. These morphological changes indicate a pronounced inhibition of F-actin polymerization (Figure 5A). In addition, the effects of astemizole and sorafenib on metastasis-related proteins were evaluated, including Integrinβ1, FAK, and MMP1, all of which are critically involved in tumor cell adhesion, invasion, and migration [30,31,32]. Treatment with either astemizole or sorafenib alone moderately decreased the phosphorylation levels of Integrinβ1 and FAK, whereas the combined treatment led to a more substantial reduction. Moreover, MMP1 expression was significantly downregulated in the combination group (Figure 5B,C). Taken together, these results demonstrate that astemizole and sorafenib synergistically inhibit HCC cell migration by disrupting F-actin cytoskeletal organization and suppressing key metastasis-associated proteins, including phosphorylated Integrinβ1, FAK, and MMP1.

### 3.7. Combination of Astemizole and Sorafenib Reduces Tube Formation by HUVECs

**Figure 6:**
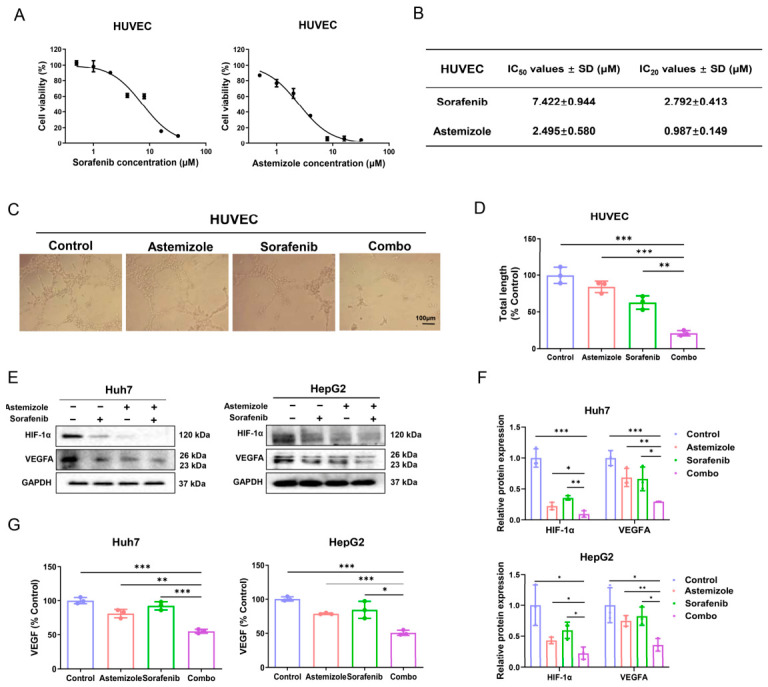
Combined treatment with astemizole and sorafenib suppresses angiogenesis in HUVECs. (**A**) Viability of HUVECs treated with increasing concentrations of astemizole or sorafenib for 48 h, as assessed by MTT assay. Concentration ranges: sorafenib, 0.5–32 µM; astemizole, 0.5–32 µM. (**B**) Summary of IC_50_ and IC_20_ values of astemizole and sorafenib in HUVECs. Sub-cytotoxic concentrations (<IC_20_) were selected for subsequent tube-formation assays (sorafenib: 2.7 µM; astemizole: 0.9 µM). (**C**) Representative images of tube formation by HUVECs cultured on matrigel following treatment with astemizole, sorafenib, or their combination. Scale bar = 100 μm. (**D**) Quantification of total tube length normalized to control. (**E**) Western blot analysis of HIF-1α and VEGFA expression in Huh7 and HepG2 cells treated with astemizole and/or sorafenib for 48 h. GAPDH was used as a loading control. (**F**) Densitometric analysis of the relative expression levels of HIF-1α and VEGFA. (**G**) VEGF concentrations in the culture supernatants of Huh7 and HepG2 cells treated with astemizole and/or sorafenib were measured using an ELISA kit. Data are presented as mean ± SD (*n* = 3). * *p* < 0.05, ** *p* < 0.01, *** *p* < 0.001.

Angiogenesis is a critical process supporting tumor invasion and metastasis [33]. To evaluate the anti-angiogenic potential of combined treatment with astemizole and sorafenib, we performed a tube formation assay using HUVECs, with the drug concentrations with less influence on cell proliferation (below IC_20_) (Figure 6A,B). It was observed that control cells formed well-developed and relatively loose capillary-like networks. Treatment with either astemizole or sorafenib alone disrupted tube integrity and reduced network density, whereas the combination of astemizole and sorafenib caused a more pronounced impairment, markedly decreasing both tubular length and branch points (Figure 6C,D). Given that angiogenesis is largely driven by VEGF signaling, and that HIF-1α is a key transcriptional regulator of VEGF expression [34], we next investigated whether the anti-angiogenic effects of astemizole and sorafenib were associated with modulation of the HIF-1α/VEGF axis. Western blot analysis revealed that both agents reduced HIF-1α and VEGFA protein levels, with the combination producing a stronger downregulation compared to monotherapies (Figure 6E,F). Furthermore, VEGF levels in the culture supernatants of Huh7 and HepG2 cells were quantified by ELISA. Consistent with the protein expression data, the combination treatment significantly suppressed VEGF secretion relative to single-agent treatments (Figure 6G). Collectively, these results indicate that the combined application of astemizole and sorafenib effectively inhibits endothelial tube formation, an effect likely mediated through coordinated downregulation of HIF-1α and subsequent reduction in VEGF expression and secretion.

## 4. Discussion

HCC is a highly lethal malignancy characterized by a high propensity for metastasis and recurrence [2]. Sorafenib, the first systemic agent approved by the FDA, provides only modest clinical benefit and is often limited by acquired resistance and adverse effects [3], highlighting the need for novel combination strategies to enhance its efficacy and reduce the toxicity. In this study, we identified astemizole, an antihistamine with reported antitumor activity, as a promising candidate for combination with sorafenib. We demonstrate that astemizole markedly sensitized HCC cells to sorafenib, leading to enhanced suppression of proliferation, metastasis, and angiogenesis. Mechanistically, the combination of astemizole and sorafenib synergistically regulated G1/S checkpoint proteins, inducing cell cycle arrest at the G1 phase. It also cooperatively modulated metastasis-associated regulators, thereby inhibiting migration, invasion, and adhesion of HCC cells. Furthermore, the dual treatment reduced VEGF secretion and downregulated HIF-1α, resulting in diminished angiogenesis. Our mechanistic findings are consistent with recent advances demonstrating that repurposing antihistamines in combination with existing targeted therapies can significantly enhance therapeutic efficacy and overcome drug resistance in HCC [35]. This emerging strategy provides a strong rationale for the synergistic potential of the astemizole–sorafenib combination.

First, we demonstrated that astemizole synergized with sorafenib to potently inhibit HCC cell proliferation, with a low-toxicity dose of astemizole significantly enhancing the efficacy of sorafenib. This synergistic growth inhibition was further confirmed by colony formation and EdU assays, indicating that the combined strategy holds potential therapeutic value. Inducing cell cycle arrest represents a key mechanism of action for many anticancer agents. Our data showed that astemizole enhanced sorafenib-induced G1 phase arrest, accompanied by downregulation of Cyclin D1 and phosphorylated Rb, along with upregulation of p27. At the G1/S checkpoint, the CDK4/6-Cyclin D1 complex promotes Rb phosphorylation, leading to E2F release and S-phase entry, whereas p27 inhibits the activity of this complex [36]. These findings suggest that the G1 arrest induced by the combination treatment may result from suppression of CDK4-Cyclin D1 complex activity. Interestingly, despite the pronounced G1 cell cycle arrest, no significant induction of apoptosis was observed, indicating that the combination of astemizole and sorafenib primarily exerts a cytostatic rather than a cytotoxic effect. Recent evidence suggests that pharmacological inhibition of the CDK4/6-Cyclin D1 axis, particularly in conjunction with p27 upregulation, drives cancer cells into a stable state of cell cycle arrest, resulting in reversible quiescence or irreversible senescence rather than apoptosis [37,38,39]. Accordingly, we speculate that, at the current combination dosages, this treatment may promote HCC cells to enter a prolonged quiescent or senescent state.

We further identified Eag1-ERK signaling as a critical node underlying this synergy. Eag1 is a recognized antitumor target of astemizole and a key regulator of the G1/S checkpoint [12,40]. Consistent with previous reports linking Eag1 suppression to reduced Cyclin D1 expression and Rb phosphorylation [40], our data show that astemizole downregulated Eag1, an effect further enhanced by combination treatment. This synergy is associated with reinforced inhibition of ERK signaling by sorafenib, likely attenuating E2F1-dependent Eag1 transcription and converging on p27 and Cyclin D1 regulation [41,42,43], thereby promoting G1 phase arrest.

Metastasis is the feature of advanced malignancies and the leading cause of cancer-related mortality. During tumor progression, cancer cells disseminate from the primary lesion through basement membrane invasion and lymphatic or vascular spread, ultimately colonizing distant organs [44]. Given the highly metastatic propensity of HCC, we further evaluated whether combined treatment with astemizole and sorafenib exerts a synergistic anti-metastatic effect. Transwell assays revealed marked suppression of cell migration and invasion following combination treatment, while adhesion assays confirmed a significant reduction in cellular adhesive capacity. Although tumor invasion requires transient attachment to the ECM to generate mechanical traction, a profound loss of adhesive capacity reflects a critical failure in the initial anchoring step of the metastatic cascade. The reduced adhesion observed in this study indicates an impaired ability of the cells to form stable focal adhesions. In the absence of this structural foundation, HCC cells are unable to generate the tension necessary for basement membrane penetration [45,46]. Consequently, severe disruption of cell–ECM interactions effectively arrests the earliest stage of invasion, thereby serving as a key indicator of the anti-metastatic potential of the combination treatment. Mechanistically, the combination modulated multiple metastasis-associated regulators. Integrin β1, a key mediator of cell–matrix adhesion, promotes tumor metastasis in part through activation of FAK [30]. FAK signaling facilitates actin cytoskeleton remodeling, focal adhesion dynamics, and MMP expression, thereby enhancing invasive behavior [31]. In addition, directional polymerization of F-actin is essential for the formation of membrane protrusions that drive cell motility [47]. Our findings suggest that the combination of astemizole and sorafenib cooperatively attenuates the migratory, invasive, and adhesive capacities of HCC cells, through suppressing Integrin β1 phosphorylation, downregulating FAK and MMP1 expression, and inhibiting F-actin polymerization.

Tumor growth is critically dependent on angiogenesis, and disruption of the balance between pro- and anti-angiogenic factors facilitates tumor recurrence and metastasis [48]. We found that astemizole significantly enhanced the anti-angiogenic activity of sorafenib in HUVECs. Among pro-angiogenic mediators, VEGF is a central driver of vascular permeability, extracellular matrix remodeling, endothelial cell migration, and tube formation [49]. VEGF expression is largely regulated by HIF-1α, a key transcriptional regulator of the tumor microenvironment [50]. Our findings indicate that the enhanced anti-angiogenic activity of the combination treatment may be attributed to suppression of HIF-1α expression, leading to reduced VEGF secretion by HCC cells.

Previous studies have demonstrated that both Eag1 and the ERK/MAPK signaling pathway have been implicated in tumor metastasis and angiogenesis [51,52]. Eag1 promotes cell migration in breast cancer by enhancing Integrin β1 phosphorylation and FAK expression, and contributes to cytoskeletal reorganization in pituitary adenoma [53]. Similarly, the ERK/MAPK pathway upregulates Integrin β1 expression and enhances MMP1 activity to facilitate invasion in chondrosarcoma [54,55]. In addition to its role in metastasis, Eag1 promotes angiogenesis through upregulation of HIF-1α and subsequent VEGF secretion [14]. Consistently, inhibition of the ERK/Elk-1 pathway has been demonstrated to suppress HIF-1α and VEGFA expression, attenuating angiogenesis in colorectal cancer [56]. Building on this evidence, we propose that the combination of astemizole and sorafenib may co-target the Eag1 and ERK/MAPK pathways, leading to coordinated suppression of Integrin β1 phosphorylation, F-actin polymerization, FAK and MMP1 expression, thereby inhibiting the metastatic and angiogenic capacities of HCC cells. Nevertheless, whether Eag1 serves as a key molecular target mediating the ability of astemizole to potentiate the anti-HCC effects of sorafenib remains to be validated through genetic manipulation. Moreover, the in vivo antitumor efficacy and safety of this combination strategy require systematic evaluation in animal models.

## 5. Conclusions

In summary, this study identifies astemizole as a promising repurposed agent that synergistically enhances the antitumor efficacy of sorafenib by coordinately targeting cell cycle progression, metastatic, and angiogenic signaling in HCC. Crucially, our results strongly corroborate and mechanistically expand upon the therapeutic paradigm recently proposed by Villarruel-Melquiades et al. [35]. While their foundational work demonstrated the translational potential of combining antihistamines with targeted therapies, our study elucidates the specific cellular phenotypic changes and underlying molecular mechanisms affected by the combination of astemizole and sorafenib, revealing that the concurrent inhibition of Eag1 and the MAPK/ERK signaling pathway may underlie its multifaceted effects. These findings provide a rationale for further conducting preclinical and translational studies to evaluate this combination in advanced HCC.

## Figures and Tables

**Table 1:** Combination indexes (CI) of astemizole and sorafenib in Huh7 and HepG2 cells.

	Drug or Drug Combination	r	Dm	CI Values		
				ED_50_	ED_75_	ED_90_
Huh7	Sorafenib	0.85814	5.97636	-	-	-
	Astemizole	0.97391	3.78462	-	-	-
	Combo	0.95977	1.03122	0.30879	0.34304	0.59109
HepG2	Sorafenib	0.98821	4.09839	-	-	-
	Astemizole	0.99073	3.94097	-	-	-
	Combo	0.99791	1.62938	0.81101	0.80294	0.83862

## Data Availability

The original contributions presented in this study are included in the article. Further inquiries can be directed to the corresponding authors.

## Abbreviations

The following abbreviations are used in this manuscript:

APS	ammonium persulfate
BSA	bovine serum albumin
CDK	cyclin-dependent kinases
DMSO	dimethyl sulfoxide
EDTA	ethylenediamine tetraacetic acid
EdU	5-ethynyl-2-deoxyuridine
ELISA	enzyme-linked immunosorbent assay
FAK	focal adhesion kinase
FBS	fetal bovine serum
HCC	hepatocellular carcinoma
HIF-1α	hypoxia inducible factor-1α
HUVECs	human umbilical vein endothelial cells
IC20	20% inhibitory concentration
IC50	50% inhibitory concentration
MMP	matrix metalloprotein
MTT	3-(4,5-dimethyl-2-thiazolyl)-2,5-diphenyl-2-H-tetrazolium bromide
PBS	phosphate-buffered saline
PI	propidium iodide
VEGF	vascular endothelial growth factor
